# SnO_2_QDs Deposited on GO/PPy-Modified Glassy Carbon Electrode for Efficient Electrochemical Hydrogen Peroxide Sensor

**DOI:** 10.3390/bios12110983

**Published:** 2022-11-07

**Authors:** Vandana Molahalli, Aman Sharma, Apoorva Shetty, Gurumurthy Hegde

**Affiliations:** 1 Department of Chemistry, CHRIST (Deemed to be University), Bangalore 560029, India; 2 Centre for Advanced Research and Development (CARD), CHRIST (Deemed to be University), Bangalore 560029, India

**Keywords:** polypyrrole, hydrogen peroxide, nanocomposite, electrochemical sensors

## Abstract

In this present work, we demonstrate an efficient electrochemical sensor for the detection of hydrogen peroxide (H_2_O_2_) using a glassy carbon electrode (GCE) modified with a ternary nanocomposite of tin oxide QDs/GO/PPy (SGP2). An in situ chemical oxidative polymerization method was used to create the SGP2 nanocomposite. FTIR, XRD, HR TEM, CV, DPV, and impedance analysis were used to characterize the nanocomposite. The SGP2 nanocomposite modified GCE can be used to create an effective H_2_O_2_ electrochemical sensor with high sensitivity and a low detection limit (LOD). With SGP2 modified GCE, the electrochemical detection test for H_2_O_2_ was carried out using cyclic voltammetry (CV) and amperometric methods. The SGP2 modified GCE shows improved sensing capabilities, resulting in considerable sensitivity of 11.69 µA mM cm^−2^ and a very low limit of detection (LOD) of 0.758 µM for a broad linear range of H_2_O_2_ concentration from 0.1 mM to 0.8 mM with a correlation coefficient R^2^ = 0.9886. Additionally, the performance of the SGP2-modified GCE electrode is on par with or nonetheless superior to that of the other functional materials that have been reported for H_2_O_2_. As a result, our findings suggest that combining conductive polymer with metal oxide may be a useful method for producing sophisticated and affordable electrochemical sensors.

## 1. Introduction

Through a recognition element that connects the analyte with the transducer and displays an electrical signal, a sensor is a device that enables the transformation of a physicochemical process into an analytical signal. To ensure that the results of any investigation are both accurate and trustworthy, a sensor’s design is crucial. Therefore, gadgets with diagnostic capabilities have been created by combining nanotechnology, electrochemistry, and analytical chemistry.

For the creation and implementation of analytical methodologies, electrochemical sensors benefit from the redox property of chemical species. The recognition element must be properly immobilised on the surface of an electrochemical sensor for it to function well, which makes the design and development of simple and compatible materials crucial. It is a developing field in nanotechnology to correctly mix organic and inorganic nanomaterials to achieve the latter goal’s with maximum efficiency.

A ubiquitous disinfectant, hydrogen peroxide is also a crucial intermediary in the biochemical, pharmacological, therapeutic, industrial, and environmental sciences. Consequently, its analytical determination plays a vital role. H_2_O_2_ is best known for its harmful effects on living things, but it also plays an important signalling role in a variety of biological processes, including immune stomata closure, cell activation, apoptosis, and root growth. H_2_O_2_ results from many metabolic processes involving the oxidase enzymes (urate oxidase, cholesterol oxidase, alcohol oxidase, glucose oxidase, amino acid oxidase) [1]. H_2_O_2_, which is produced by a subclass of white blood cells known as neutrophils, serves as the first line of defence in the human body against toxins, parasites, bacteria, viruses, and yeast. H_2_O_2_ is also important in regulating renal function and acting as an antibacterial agent in urine [2]. In addition to irritating the skin, eyes, stomach, mouth, and intestines, high levels of H_2_O_2_ in blood plasma might be a pathogenic element in the damage to vascular organs brought on by systemic hypertension [3]. In light of all of these, the thorough examination and detection of H_2_O_2_ are simply essential for both academic and industrial purposes. The electrochemical method is particularly competitive and appealing among the different techniques created for the quantitative detection of H_2_O_2_ due to its benefits, such as simple operation, easy downsizing, low cost, high sensitivity, strong stability, and the potential for real-time detection [4]. The two basic types of electrochemical H_2_O_2_ sensors are enzymatic and non-enzymatic sensors. Researchers have used enzyme-based electrochemical H_2_O_2_ sensors as the fastest of the two established sensor approaches due to their outstanding electrochemical transduction process sensitivity and excellent selectivity of the biological recognition elements. There are several limitations to enzymatic sensors, including high costs due to the expensive materials used, confined activity and storage time, a difficult and complicated immobilisation process, and low replicability. In light of these constraints, non-enzymatic H_2_O_2_ electrochemical sensors with no standing properties have recently received much attention. The non-enzymatic method provides several benefits over the enzymatic sensor, including ease of use, inexpensive prices, high reproducibility, improved stability, and high selectivity and sensitivity [3,5]. Several strategies were looked into in order to reduce the high over potential and improve the current response of H_2_O_2_. Utilizing modified electrodes made of materials with advantageous qualities is one of the possible methods for overcoming the drawbacks [6,7,8]. Additionally, nanomaterials like noble metals, metal oxides [9,10], and alloys [11] were frequently used to create non-enzymatic H_2_O_2_ sensors in order to improve sensor performance. This can be attributed to their unique benefits of having a large surface area, excellent catalytic properties, and enhanced mass transport.

A lot of research has been done on the H_2_O_2_ sensors based on metal oxide nanoparticles as Fe_3_O_4_, MnO_2_, SnO_2_, WO_3_, CuO, and Co_3_O_4_ because of their peculiar structural properties and large surface areas [12]. The metal oxide is frequently combined with conducting materials like carbon nanotubes or graphene to achieve high sensitivity. However, graphene often experiences irreversible aggregation and is challenging to synthesize, whereas carbon nanotubes are expensive. In order to create simple, susceptible H_2_O_2_ sensors, a new conducting material must be created that can support metal oxide nanoparticles. Additionally, conducting polymers like polyaniline, polypyrrole, polythiophene, and their derivatives have a variety of uses in electrochemical sensors [13]. Among these conducting polymers, polypyrrole (PPy) is a well-studied -electron conjugated conducting polymer due to its good environmental stability, high electrical conductivity in ambient conditions, and less toxicological issues [14]. There is currently a lot of interest in creating hybrid nanocomposite materials by mixing metal oxides with conducting polymers that perform better than their equivalents. The remarkable features of these nanocomposites were demonstrated in a variety of applications, including sensors, solar cells, supercapacitors, and other organic electronics [15,16]. On the other hand, due to their excellent electrical and electrochemical properties, tin oxide quantum dots (SnO_2_QDs), a wide bandgap semiconductor, have received a lot of interest for their numerous uses in electronics and optics. According to a recent literature review, the tin oxide nanostructure has been researched as an important material due to its novel features and functionality caused by the quantum confinement effect [17,18]. Many methods for producing tin oxide (SnO_2_) nanocrystal have been devised, including spray pyrolysis, fast oxidation of elemental tin, oxide powder thermal evaporation, tin grain evaporation in air, chemical vapour deposition, sol-gel approach [19] and hydrothermal procedures [20], but less research has been done on SnO_2_ quantum dots.

Recently, a single layer of sp^2^ hybridized carbon atoms called graphene has received much attention in developing advanced materials. The first steps in producing graphene oxide (GO), a single layer of graphite oxide, involved treating graphite flakes with potent aqueous oxidizing agents and epitaxial chemical vapour deposition [21]. Due to its unique structure, high solution-processability, and ease of post-functionalization of GO, this wonderful material has a wide range of applications in various fields. Graphene has found uses in the production of electrochemical sensors, capacitors and biosensors because of its excellent conductivity [22,23,24].

In recent years, various types of nanostructured PPy combined with graphene, GO, or reduced GO (rGO) nanosheets have been prepared and used to fabricate electrodes due to polypyrrole’s distinctive properties, including its higher conductivity, low cost, excellent thermal stability, rapid charge–discharge mechanism, and high energy density [25]. According to Gurusamy et al. [4], the electrochemical measurement of hydrogen peroxide (H_2_O_2_), using a modified glassy carbon electrode (MWCNT/(BIM-Cu^2+^)_n_@GCE) coordinated with poly(bisbenzimidazol at copper(II)), was proposed by Gurusamy et al. This form of coordination polymer web for peroxide sensing is documented. The electrode demonstrated an H_2_O_2_ detection limit of 7.8 × 10^−7^ M and a sensitivity of 5 nA μM^−1^ in amperometric sensing.

In this study, SGP2 ternary composites (polypyrrole, tin oxide quantum dots, graphene oxide, in the ratio 1:1:2 coded as SGP2) were made utilizing an in situ chemical oxidation approach, and tin oxide quantum dots were created using a conventional hydrothermal process, and graphene oxide was synthesized by the modified Hummer’s method. To describe the oxidation state, chemical composition, and electrocatalytic capabilities of the SGP2 ternary material using several techniques such as Fourier Transform Infrared Spectrometer (FTIR), Transmission Electron Microscope (TEM), X-Ray diffraction (XRD), Cyclic Voltammetry (CV), Difference Pulse Voltammetry (DPV), Atomic Force Microscopy (AFM), and Electrochemical Impedance Spectroscopy (EIS) studies are applied. We looked at the ternary composites’ optical, electrical, and sensor studies. The present ternary nanocomposite (SGP2) with higher sensitivity of 11.69 µA mM cm^−2^ and low limit of detection 0.758 µM is a better material for the H_2_O_2_ sensor. The modification of the sensor electrode utilizing graphene oxide-decorated SnO_2_ quantum dots/polypyrrole ternary composites has been developed to enhance the sensing capabilities of H_2_O_2_ sensors.

## 2. Materials and Methods

### 2.1. Chemical Reagents

Pyrrole monomer (C_4_H_5_N) (99%, Sigma-Aldrich, Bengaluru, India), Ferric chloride (FeCl_3_) (97%, Sigma-Aldrich, Bengaluru, India), Tin (IV) chloride pentahydrate (SnCl_4_·5H_2_O) (98%, Sigma-Aldrich, Bengaluru, India), Hydrazine hydrate (N_2_H_4_·H_2_O) (98%, Merck Chemicals, Bengaluru, India), graphite powder (99.99%, Sigma-Aldrich, Bengaluru, India), and Polyvinylidene difluoride (PVDF) (99%, Sigma-Aldrich, Bengaluru, India) were used without further purification. Potassium Hydroxide (KOH), N-Methyl-2-pyrrolidone (NMP), Hydrogen peroxide (H_2_O_2_), and activated carbon were purchased from Merck Chemicals, Bengaluru, India. Nitric acid (HNO_3_) and Sulphuric acid (H_2_SO_4_) were purchased from Merck Chemicals, Bengaluru, India (99.99%), in addition to Polyvinyl Alcohol (PVA) being obtained (99%, Sigma-Aldrich, Bengaluru, India). All the reagents were used as received without any purification. Ultra-pure water was used throughout the experiments, which was attained from a Milli-Q system from Millipore (Milford, MA, USA).

### 2.2. Synthesis of SnO_2_ Quantum Dots

The 1.9 g of N_2_H_4_. H_2_O (0.3 M) and 3.79 g of SnCl_4_·5H_2_O (0.1 M) were mixed together in 200 mL of distilled water with constant stirring. SnCl_4_ reacted immediately with hydrazine hydrate (N_2_H_4_. H_2_O) to form a slurry-like white precipitate. After stirring for 20 min, the solution was placed in a Teflon-lined autoclave fixed at 180 °C for 24 h. SnO_2_QDs were created by centrifuging the mixture for 30 min at 3000 rpm, rinsing it with distilled water, and drying it for two hours at 50 °C.

### 2.3. Synthesis of Graphene Oxide

The modified Hummer’s method was used for the synthesis of GO. In a conical flask held at 0 °C with continuous stirring, 1.5 g of NaNO_3_, 1.5 g of graphite powder, and 23 mL of concentrated H_2_SO_4_ dissolved in 500 mL of deionized water were mixed. In addition, 2.5 g of KMnO_4_ were added to the flask and stirred for 3 h. Dropwise, 46 mL of deionized water were added to the flask and heated at 60 °C for 2 h with constant stirring. With constant stirring for an hour, 10 mL of H_2_O_2_ were added to the flask. Following the change in colour of the solution, the suspension was filtered and washed several times with deionized water before drying for 2 h at 50 °C.

### 2.4. Synthesis of SGP2 Ternary Composite

**Scheme 1:**
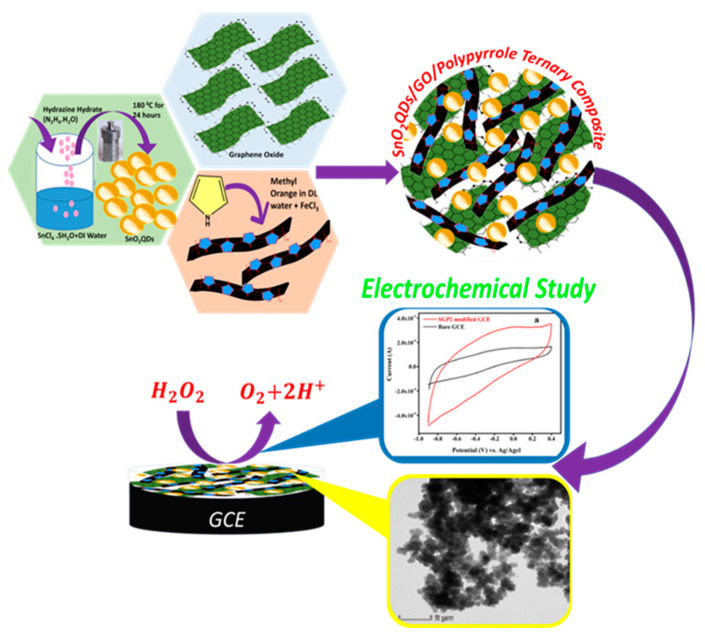
Synthesis of SnO_2_QDs/GO/PPy ternary composites for electrochemical sensing applications.

The SGP2 ternary composite was created using in situ polymerization of pyrrole with HCl and FeCl_3_ as an initiator. Drop-by-drop additions of SnO_2_ QDs and GO were made after 2 mL of pyrrole monomer were added to 1 M HCl (100 mL) and agitated for an hour. After being sonicated for 30 min, the above-mentioned suspension/solution was kept at 0 °C for 24 h. The pyrrole suspension was then kept at 0 °C with constant stirring for a day to allow for polymerization, and then 11.354 g of FeCl_3_ were added drop-by-drop. A ternary composite powder made of SnO_2_ QDs, graphene oxide, and PPy was formed after the suspension was centrifuged for 15 min at 3000 rpm. The schematic representation of synthesis of SGP2 ternary composite for electrochemical sensor was shown in Scheme 1.

## 3. Characterization Techniques

The Fourier Transform Infrared Spectrometer (FTIR) model ATR ALPHA BRUKER and X-ray diffractometry (XRD) by Rigaku Miniflex-II (Tokyo, Japan) with Cu Kα radiation of wavelength λ = 1.5406 A° at a scan rate of 5° min^−1^ with 2θ values from 2° to 80° were used to study the chemical change. The Transmission Electron Microscope was used to investigate the TEM (model JEOL, JEM-2100, Peabody, MA, USA), with the Schottky type field emission gun as an electron source, with nano and convergent beam modes as operating modes (Operating HT 80–200 kV), with lattice resolution as 0.1 nm. The CH166E electrochemical workstation was used to record the electrochemical measurements. The dielectric study was conducted by using the Wayne kerr 6500 B series Impedance Analyser, Hemer, Germany.

## 4. Results and Discussion

### 4.1. FTIR Study

**Figure 1:**
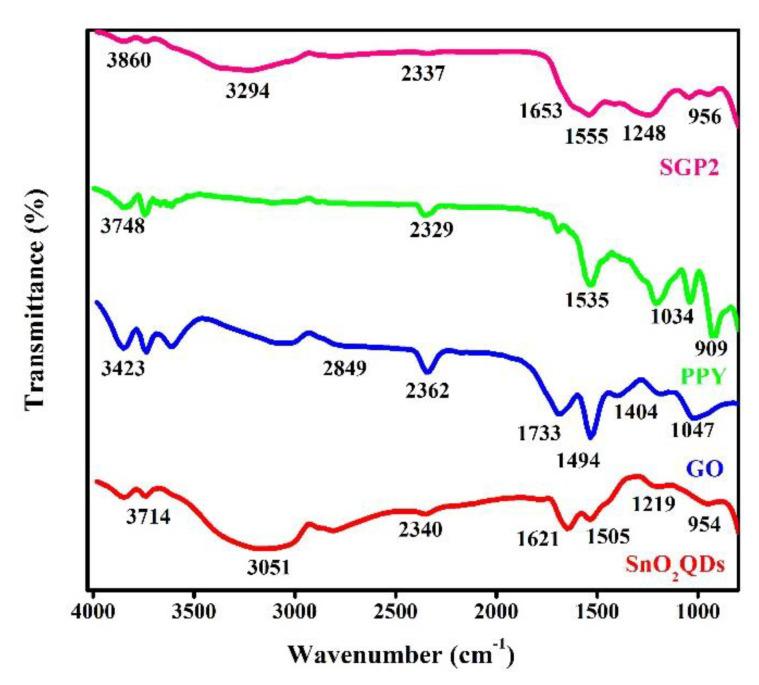
FTIR spectra of pure SnO_2_ QDs, GO, PPy, and SGP2 composite.

In order to create a SnO_2_ quantum dots hybrid complex, hydrazine hydrate interacts with tin chloride. This interaction results in vibrational bands in the FTIR spectra of SnO_2_ QDs shown in Figure 1. The N-H is attributed to the peaks at 954 cm^−1^ that result from bending vibration. The twist and wag bands at 1505 cm^−1^ and 1219 cm^−1^, respectively, are evident between two metal ions. The O-H vibration of the absorbed water contributes to the peak at 1621 cm^−1^. The peaks at 2340 cm^−1^, 3051 cm^−1^, and 3714 cm^−1^ are evidence that SnO_2_ quantum dots are in their crystalline form [26].

A distinctive peak at 3423 cm^−1^ caused by the O-H group’s presence in the GO. The peaks at 1733 cm^−1^ and 1047 cm^−1^, respectively, indicate the COOH (carboxy) and C-O-C (epoxide). In addition, 1494 cm^−1^ represents the remaining sp^2^ carbon in graphite. The PPy has two bands: one at 1034 cm^−1^, which corresponds to the stretching vibration of the C-N in-plane ring vibration with high conjugation, and one at 1535 cm^−1^, which corresponds to the C-C bending vibration [27]. Furthermore, =C-N out of plane deformation is represented by the band seen near 909 cm^−1^ [28]. The formation of polypyrrole is confirmed by the band at 3748 and 2329 cm^−1^, which is due to the N-H and C-H stretching vibrations [29]. The peak of SGP2 at 956 cm^−1^ can be attributed to the ring stretching mode of PPy present in the composite and 1653 cm^−1^ assigned from the O-H stretching of SnO_2_ present in the composite. Therefore, FTIR results show the presence of SnO_2_, GO, PPy in the ternary SGP2 composite.

### 4.2. XRD Analysis

**Figure 2:**
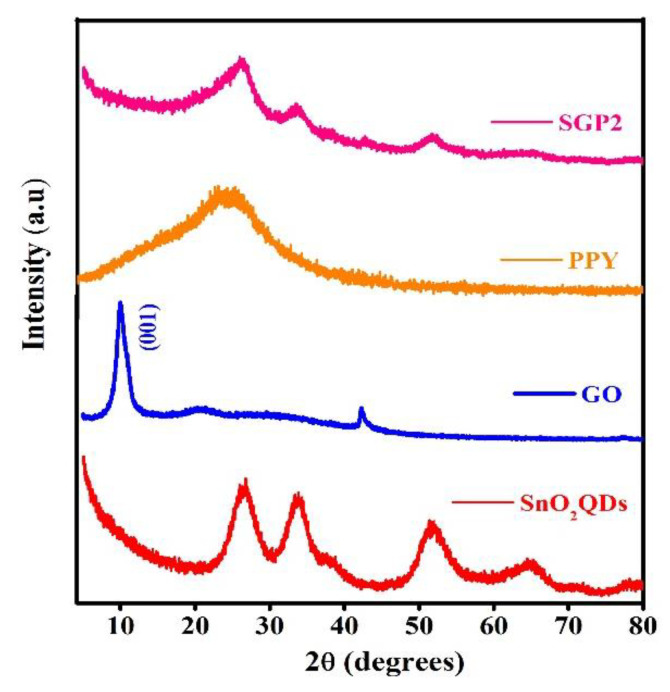
X-ray diffraction (XRD) patterns for the SnO_2_ QDs, PPy, GO, and SnO_2_ QDs/GO/PPy composites.

The XRD analysis depicted in Figure 2 was used to examine the polycrystalline phase of SnO_2_ QDs. All the peaks indexed in the SnO_2_ QDS were comparable with ICDD No. 41-1445 without the presence of impurities [30]. The average crystallite size was calculated by using the Scherrer equation,

L = kλ/βcosθ, where crystallite size (L), λ is the wavelength of Cu-kα radiation 1.5406 A°, θ the Bragg diffraction angle (in radian), and β the integral breadth (i.e., area under the curve divided by maximum height, in radian). The average crystallite size of SnO_2_ QDs obtained is 2.78 nm, which is well in agreement with the TEM data.

The graphene’s oxygen functional group is indicated by the large diffraction peak at 2θ = 9.78° with (001) crystal plane [31]. The small peaks at 2θ = 20° and 42° indicate that graphene and an oxygen atom are not completely linked. The broad peak at approximately 2θ = 24.63° is responsible for the polypyrrole’s amorphous character [28]. The SGP2 composite exhibits all the distinctive peaks of SnO_2_ QDs, GO, and PPy.

The polymer and SnO_2_ QDs do not aggregate on the graphene oxide surface, and they are evenly distributed throughout the SGP2 composite, as seen by the broad peak at 2θ = 26.2°, which represents the polymer present in the composite.

### 4.3. TEM Analysis

**Figure 3:**
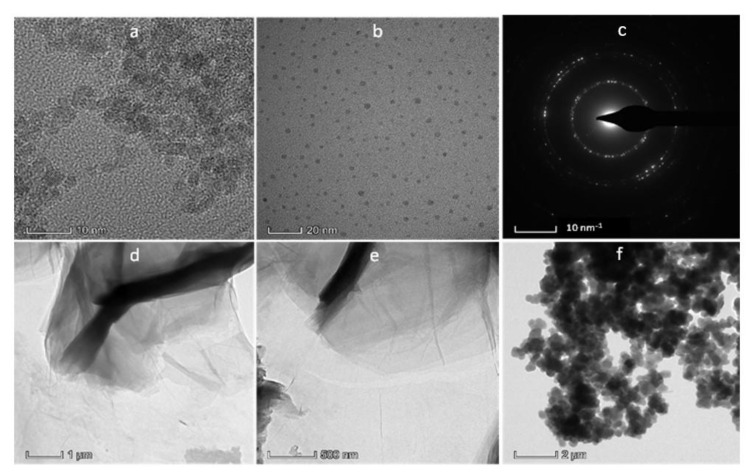
TEM analysis of SnO_2_ QDs (**a**,**b**) SnO_2_QDs; (**c**) SAED pattern of SnO_2_ QDs; (**d**,**e**) TEM image of GO; (**f**) SGP2 composite.

SnO_2_ QDs are depicted in Figure 3a,b in their respective High Resolution-TEM and TEM images, and Figure 3c depicts the corresponding selected region electron diffraction patterns, which clearly demonstrate lattice fringes. The lattice spacing of around 0.33 nm was used to study the tetragonal crystal structure of SnO_2_ QDs, as illustrated in Figure 3a.

In hydrothermal settings, the reaction temperature, reaction time, and solution pH all affect the size morphology of the QDs. The SnO_2_ quantum dots change into larger sizes with a cubic shape when the pH value is altered [32].

Figure 3b exhibits spherical SnO_2_ QDs with particle sizes ranging from 2 to 5 nm that were produced following a 24-h heating period at 180 °C and a pH of 10. The well-crystallized state of the rutile tetragonal SnO_2_ QDs is confirmed by the SAED pattern in Figure 3c. The TEM image of graphene oxide shows the transparent graphene sheet with a thin-film structure that is the result of the rapid elimination of intercalated oxygen caused by the exfoliation or sonication technique used to eliminate the multi layered disorganized graphene sheets shown in Figure 3d,e [33]. Figure 3f depicts the black dots array of SnO_2_ quantum dots distributed across the graphene sheet as a result of the SnO_2_ QDs and PPy’s strong electrostatic attraction to one another. In the SGP2 nanocomposite, SnO_2_ QDs were dispersed into the GO and PPy layers. The SnO_2_QDs are efficiently formed on the graphene nanosheets’ surface, and these thin graphene layers can significantly increase the composite’s electrical conductivity. Additionally, the graphene sheets’ individual SnO_2_QDs can speed up the diffusion of the electrolyte away from the electrode. When the structures are utilized as electrochemical electrodes, restacking of the graphene layer renders the final material less porous and electrolyte infiltration more challenging. The attachment of SnO_2_QDs may prevent the restacking of graphene layers in composites. Electrochemical studies have proven that the intercalation of SnO_2_QDS improves the material’s cyclic stability by facilitating the transport of electrolyte ions into electrodes.

### 4.4. Fabrication of Sensor and Electrochemical Study

The glassy carbon electrode (GCE) was sonicated with water and ethanol before being polished with an alumina paste of 0.5 μM and 0.05 μM prior to electrode modification. Following thorough cleaning and polishing, the GCEs were changed by coating them with SGP2 active material and Polyvinylidene fluoride binder; 3 μL of SGP2 composite solution were drop-casted on the surface of clean GCE having 0.07 cm^2^ active surface electrode area and allowed to adsorb upon drying in a vacuum oven. The CHI 660E electrochemical workstation was connected to a standard three-electrode setup with platinum wire as the counter electrode, Ag/AgCl as the reference electrode, and SGP2 modified GCE as the working electrode. For SGP2 modified GCE, several H_2_O_2_ concentrations ranging from 0.1 to 5 mM were used in the experiments.

**Figure 4:**
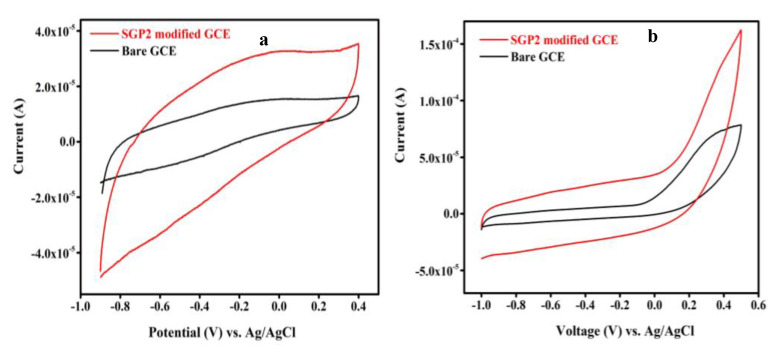
Cyclic voltammetry curves for (**a**) the absence of H_2_O_2_ and (**b**) in the presence of 0.1 mM H_2_O_2_.

All electrochemical tests for the detection of H_2_O_2_ were carried out at room temperature with continuous magnetic stirring in a 0.1 M Phosphate Buffered Saline (PBS) (pH 7.4) solution. The CHI 660E electrochemical workstation was used for all electrochemical measurements. To compare the electrocatalytic activity of bare GCE and SGP2 modified GCE, cyclic voltammetry (CV) tests were conducted in 0.1 M PBS solution both with and without H_2_O_2_. Figure 4a demonstrates the CVs of unmodified GCE and GCE modified with SGP2 at a scan rate of 30 mVs^−1^ in 0.1 M PBS solution without H_2_O_2_.

Figure 4a, in contrast to SGP2 GCE, which displays a peak current response of roughly 36 mA, the observed bare GCE and SGP2 GCE of the CV curve demonstrate the absence of analytes. In Figure 4b, bare GCE exhibits minimal electrocatalytic activity for the detection of H_2_O_2_, while SGP2 GCE exhibits a prominent oxidation peak in the presence of H_2_O_2_ analyte and a maximum peak current response of 160 mM, which is a four times higher current rate than in the absence of analyte. Due to the functionality of graphene and oxygen, SGP2 GCE is quickly oxidized.

**Figure 5:**
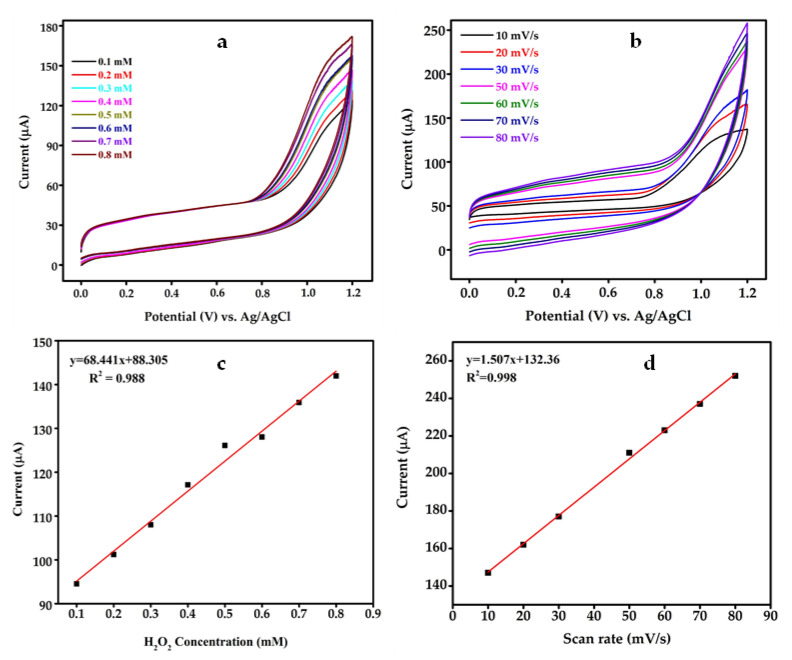
(**a**) Cyclic voltammograms curve of SnO_2_ QDs/GO/PPy ternary composite modified GCE measured in 0.1 M PBS (pH 7.4) containing different concentrations of H_2_O_2_; (**b**) CV curve of SGP2 modified GCE at different scan rates; (**c**) the plot of peak current vs. concentration of H_2_O_2_ (**d**) the plot of peak current vs. scan rates.

CV curve in Figure 5a was measured in the presence of various concentrations of H_2_O_2_ from 0.1 mM to 0.8 mM with PBS electrolyte. As the concentration of analytes was increased, the oxidation peak gradually moved toward the higher current rate, and the CV curve maintained its shape at the higher current rate, indicating the high electrocatalytic activity in the fabricated SGP2 GCE. Figure 5b shows the SGP2 CV curve measured at various scan rates from 10 mV s^−1^ to 80 mV s^−1^ in the presence of H_2_O_2_. Since the peak current rate increased as the scan rate was changed, the CV curve remained the same shape while the oxidation current only changed, and the SGP2 GCE has good electro-active material for sensor applications. Figure 5c shows the SGP2 GCE’s calibration curve when H_2_O_2_ is present. As the H_2_O_2_ concentration increases, the oxidation peak current becomes linearly proportional to the analyte species, yielding a regression value of R^2^ at ~0.988.

The SGP2 GCE calibration plot in Figure 5d demonstrates that the scan rate is being increased, and the peak current is moving toward a higher current rate. The manufactured electrode also shows that the scan rate effect is good, with an observed regression value of R^2^ at ~0.998.

### 4.5. Differential Pulse Voltametry Analysis of SGP2 GCE

**Figure 6:**
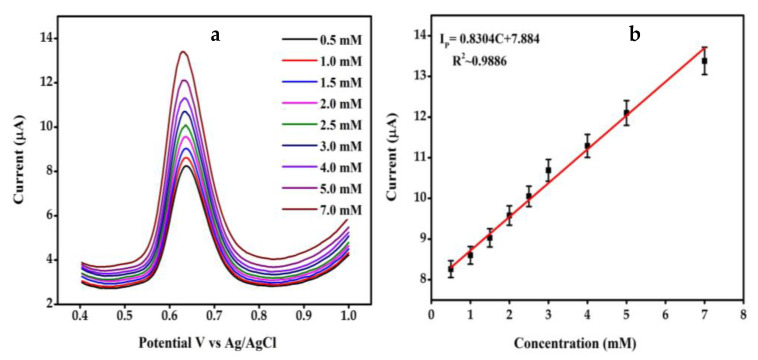
(**a**) DPV plot of SGP2 GCE and (**b**) linear calibration plot.

Figure 6a shows the DPV curve of the SGP2 GCE in the presence of various concentrations of H_2_O_2_ analyte. As hydrogen peroxide was continuously added to the PBS electrolyte at concentrations ranging from 0.5 mM to 7.0 mM, the oxidation peak current rate increased. As a result, the SGP2 GCE quickly detected the analyte and provided a high current rate because of the layer-structured GO. Figure 6b of the SGP2 GCE depicts the calibration plot as a function of H_2_O_2_ concentration. The calibration plot demonstrates the peak current rate versus concentration of H_2_O_2_, and the archived linear Equation (1) is shown [34].

I_P_ = 0.8034C + 7.884; (R^2^ = 0.9886) (1)

The SGP2 GCE exhibits a high sensitivity of 11.69 µA mM cm^−2^ and a low limit of detection (LOD) of 0.758 µM; these results suggest that the archived value is higher than the previously reported results as mentioned in literature [35,36,37]. In Table 1, a comparative study of the analytical performance of the proposed SGP2 modified GCE sensor with the previously published sensors for H_2_O_2_ detection is presented [4,38,39,40,41]. To the best of our knowledge, the proposed SGP2 modified GCE has a suitable sensor electrode for various environmental and clinical diagnostic applications.

### 4.6. Electrochemical Impedance Spectroscopy (EIS)

**Figure 7:**
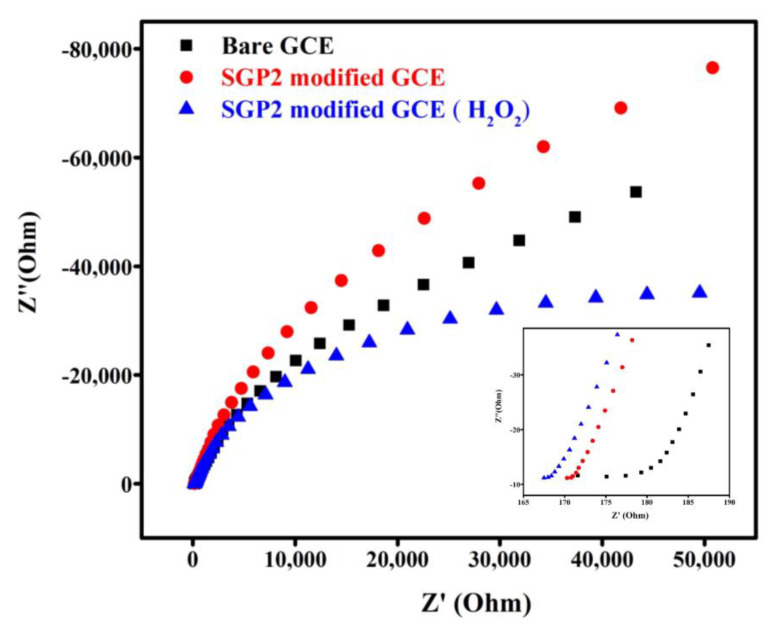
EIS analysis of bare GCE, SGP2 modified GCE, and SGP modified GCE in the presence of H_2_O_2_.

The properties of the fabricated electrode interface are further analyzed using EIS. A typical Nyquist plot has a semicircle segment that represents the electron-transfer resistance (R_ct_) at higher frequencies and a linear section that represents the diffusion limiting process at lower frequencies. The Warburg resistance (ZW), which is caused by frequency-related ionic diffusion in the electrolyte and electrode surface, represents the slope area. The impedance plot at low frequency should be linear as a result of the presence of a “constant phase element”. Figure 7 depicts the charge transport procedures in the H_2_O_2_-containing SGP2 modified electrode, doped GCE electrode, and bare GCE electrode. To understand the process of electron transfer, the charge transfer resistance (R_ct_) at the electrode-electrolyte interface is computed. The dielectric and insulating properties of the electrode affect the value of R_ct_.

R_ct_ is a crucial factor in determining the coated electrode material’s effectiveness in transferring charges from a solution to an electrode. It has been noted that SGP2-doped GCE in the presence of H_2_O_2_ has an R_Ct_ value of 168.32 while bare and SGP2-doped GCE have R_ct_ values of 180.40 and 171.01, respectively. This implies that getting a better sensor response is possible when PPY/GO and SnO_2_ are present in the composite. SnO_2_ QDS’s greater conductivity improves the kinetics of charge transfer. The EIS results are accompanied by the CV data, which showed that the SGP2 nanocomposite in the presence of H_2_O_2_ exhibited higher peak current.

### 4.7. Selectivity Study SGP2 Glassy Carbon Electrode

**Figure 8:**
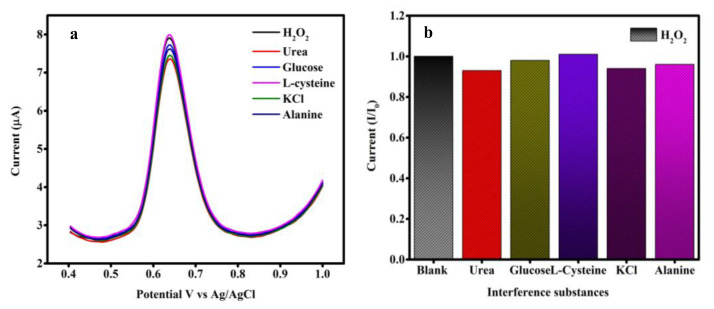
The interference of SGP2 modified GCE for H_2_O_2_ (2 mM) was evaluated in the presence of different interference substances shown in both DPV (**a**) and bar graph (**b**).

Interference study is a useful method to check the selectivity of developed electrochemical sensor towards H_2_O_2_ detection based on SGP2 nanocomposites. Examination of the interference of SGP2 modified GCE for H_2_O_2_ was determined in the presence of 2-fold higher concentration of various interferences substances like urea, glucose, L-cysteine, KCl and alanine. The DPV curve (Figure 8a) shows the no extra peak observed in the figure, which indicates that, with the addition of different interference substances, no change in the DPV curve proves that the fabricated sensor had good selectivity towards the H_2_O_2_ determination. Figure 8b shows that the oxidation peak current of (I/Io) was examined by the presence of interference (I) and absence of interference (Io); this clearly shows that the interference substance has no effect on the H_2_O_2_ and an achieved RSD of less than 1%. A fabricated SGP2 modified GCE sensor is suitable for various clinical diagnosis applications.

### 4.8. AFM Analysis

**Figure 9:**
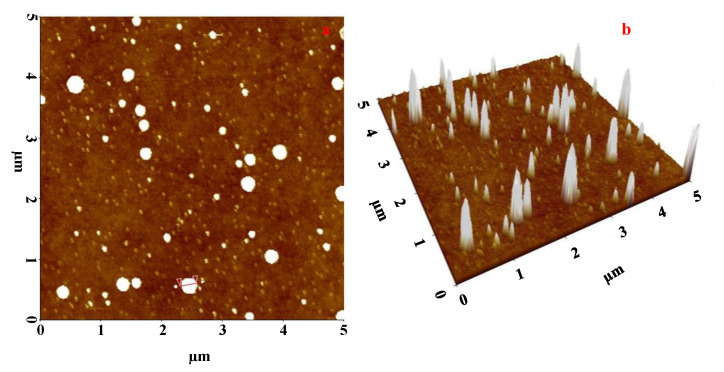
AFM analysis of (**a**) 2D image of SGP2 and (**b**) corresponding 3D images.

To evaluate the structural morphology and surface roughness of the SGP2 composites by AFM investigation (Figure 9), AFM images of the SGP2 composite show a spherical morphology, and AFM 3-D micrograph images of the SGP2 show a uniform and homogeneous morphology with high prominence, with an average roughness obtained from the topological study of 1.863 nm and a particle size of 0.3 to 5 nm. This result indicates the compatibility of the polymer matrix with nanoparticles [42].

### 4.9. Dielectric Study of SnO_2_, GO, SGP2 Composite Film

**Figure 10:**
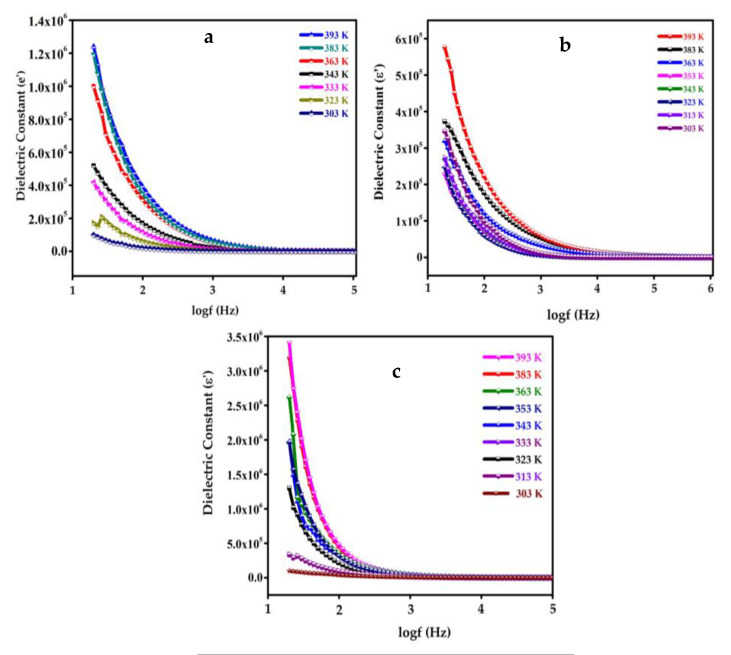
Variation in dielectric constant (ε′) with frequency at different temperatures for (**a**) SnO_2_ QDs, (**b**) GO, and (**c**) SGP2 composite film.

With a complex dielectric constant, the real and imaginary parts of the dielectric properties are typically examined using the equation ε* = ε′ − I ε″, where ε″ and ε′ are the imaginary and real parts, respectively [38]. In the presence of an external field, it releases stored and lost energy. The dipole moment polarizability is related to the dielectric constant shown in Figure 10. The plots of the frequency-related dielectric constant and dielectric loss (ε′ and ε″) demonstrate that the dielectric behaviour of polymer composites depends on a lower frequency and is independent of a higher frequency. Utilizing capacitance Cp and loss factor tan δ, the real and imaginary components of dielectric properties are computed using the Equations (2) and (3):

ε′ = Cp d/(ε^0^ A) (2)

ε″ = ε′ − tan δ(3)

**Figure 11:**
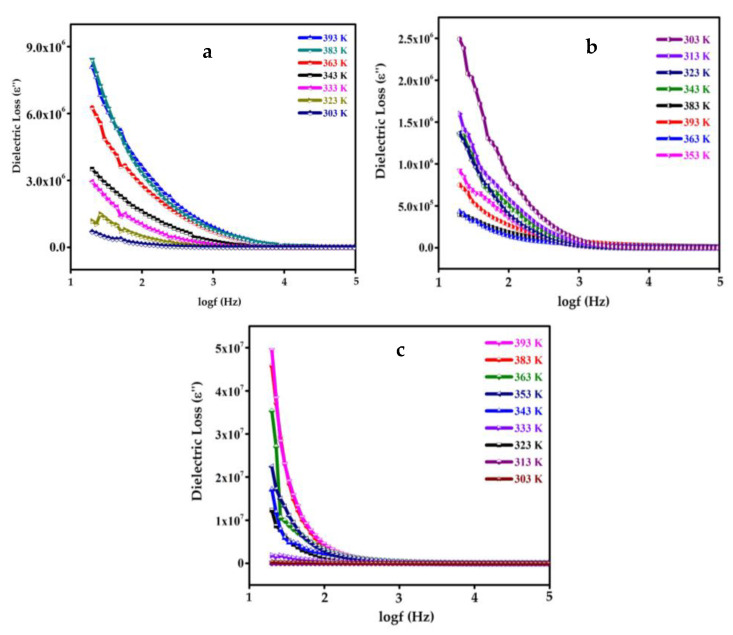
Dielectric loss (ε″) variation with frequency at different temperatures for (**a**) SnO_2_ QDs, (**b**) GO, and (**c**) SGP2 composite film.

Lower frequency increases owing to the energy accumulation between the polymer composite and the electrode. However, charge carrier density increases at high temperatures due to ion aggregation dissociation, which, as a result, increases both ε′ and ε″ shown in Figure 10 and Figure 11. Thereby, due to chain scissioning in polymer composites, the increasing trend of ε′ at various temperatures indicates the generation of bandgap defects [43]. At low frequencies less than 100 Hz, the dielectric constant of all pure SnO_2_, GO, and SGP2 composites increased dramatically.

When compared to comparable polymer nanocomposite values in the literature, the dielectric constant for SnO_2_/GO/PPy ternary composites (SGP2) is in the region of 3.5 × 10^6^, which is high [44]. This might be because the composite contains GO and SnO_2_ nanocrystals. This suggests that the substantial polarization delocalization [45] may be connected with the high dielectric response of SGP2 composites at low frequency (100 Hz–1 kHz). In comparison to pure SnO_2_ film, the ε′ and ε″ values of SGP2 composites are 2 to 3 times greater. Thus, the results demonstrate temperature-dependent variations of the dielectric parameters. All of the values rise with temperature, and for SGP2, as depicted in Figure 10 and Figure 11c, the values are maximum at a certain temperature. This is explained by the interfacial polymerization of SGP2 composites from the external field at high temperatures. As a result, the ternary polymer composite is an effective material for studying how composites behave electrically.

## 5. Modulus Study

To better understand the dielectric process, use the electric modulus M* = M′ + iM″, where M′ and M″ are real and imaginary components of the complex dielectric modulus M*, which may be computed from the ε′ and ε″ using the Equations (4) and (5) [46]:

M′ = ε′/[(ε′)^2^ − (ε″)^2^] (4)

M″ = ε″/[(ε′)^2^ − (ε″)^2^] (5)

**Figure 12:**
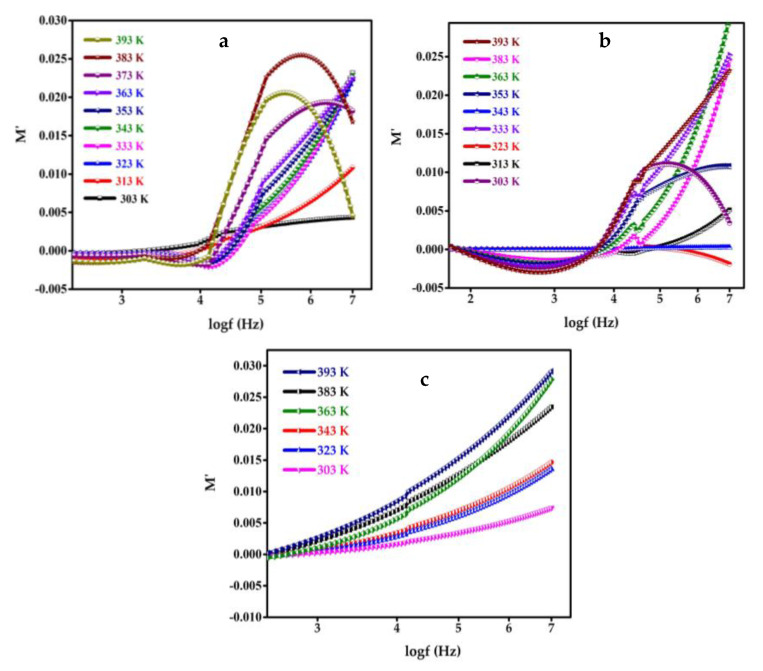
Electric modulus (M′) variation as a function of frequency at different temperatures for (**a**) SnO_2_ QDs, (**b**) GO, and (**c**) SGP2 composite film.

Figure 11 and Figure 12 depict the fluctuations in the real and imaginary components of the electric modulus M′ and M″ of ternary composites made of SnO_2_, GO, and SGP2 as a function of frequency in the 20 Hz to 1 MHz range.

**Figure 13:**
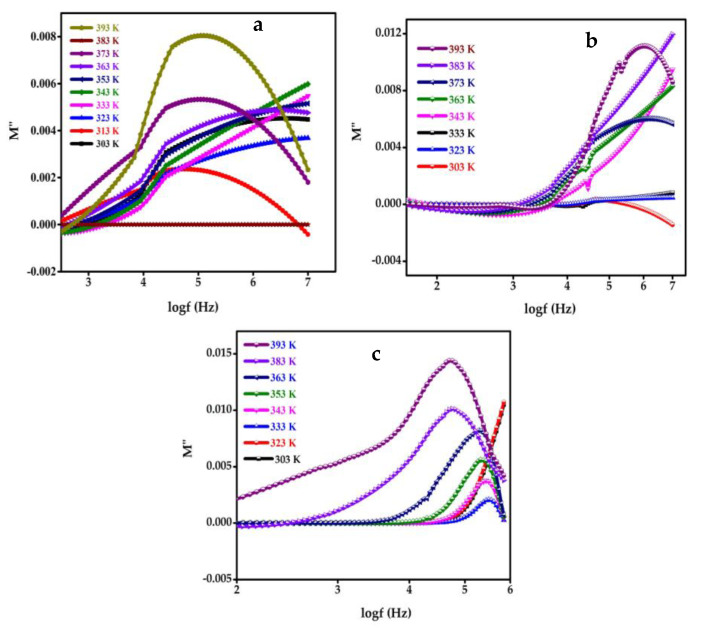
Electric modulus (M″) variation as a function of frequency at different temperatures for (**a**) SnO_2_ QDs, (**b**) GO, and (**c**) SGP2 composite film.

Because of the suppression of electrode polarization at low frequencies, M′ approaches zero; however, M′ increases with frequency, indicating that the restoring force determines the mobility of charge carriers when an electric field is present. Due to the charge carrier’s short-range mobility and the conduction electrons’ storage mobility, M″ reaches a maximum value of 1.46 × 10^−2^ as illustrated in Figure 13c.

**Figure 14:**
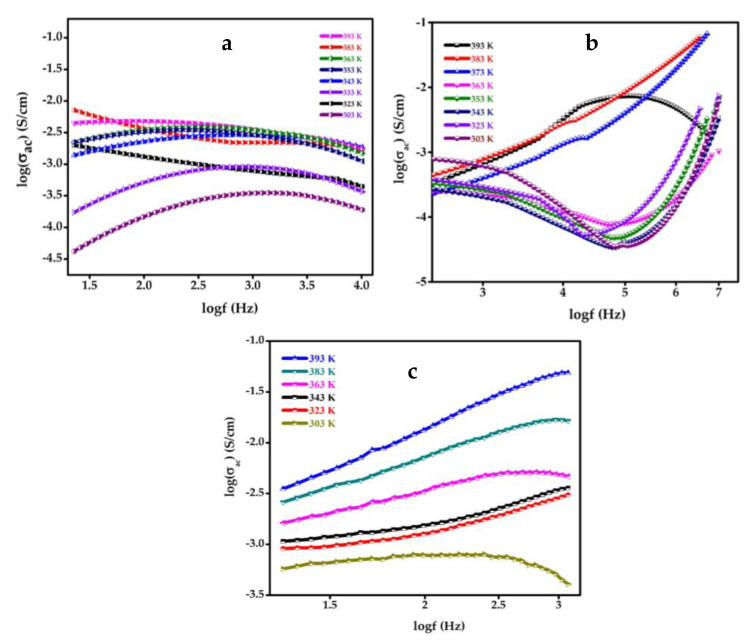
Variation in AC conductivity for (**a**) SnO_2_ QDs, (**b**) GO, and (**c**) SGP2 composite film at different temperatures.

Figure 14 shows the AC conductivity of the ternary composites of SnO_2_, GO, and SGP2. For SGP composites, the critical frequency is 2 kHz, after which there is a modest rise in conductivity with frequency but no appreciable change in conductivity.

Conductivity increases linearly with frequency and temperature beyond 2 kHz, and because of the free charges in the chain, it reaches the charge carrier’s vibration frequency together with the polymer at this point. Due to active trapped charges, conductivity is almost independent at low frequencies and reliant on high frequencies. The AC conductivity was calculated by using the Equation (6) [47],

σ_ac_ = ωc_p_d tan δ/A (6)

where A is the electrode area, d is the thickness of the sample, and ε_0_ is the dielectric permittivity in free space (8.85 × 10^−12^ F/m). According to Figure 14, the conductivity rises with temperature and frequency. The conductivity of the ternary composite material is higher than that of pure SnO_2_ and GO. The σ_ac_ values for SnO_2_, GO and SGP2 are −2.2 S/cm, −1.294 S/cm, and −1.07 S/cm, respectively. The higher chain order of the polymer composite caused by the significant intercalation between the pure SnO_2_QDs and GO with PPy is attributed to the SGP2’s maximum value agreement with dielectric data and conductivity. As the SnO_2_ QD concentration rises, the ions in the polymer have more flexibility, which enhances conductivity.

## 6. Conclusions

In conclusion, SGP2 nanocomposites synthesized utilizing an in situ chemical oxidative polymerization process are used to manufacture a newly created and efficient hydrogen peroxide electrochemical sensor. The produced nanocomposites were examined utilizing impedance analysis, FTIR, XRD, and AFM. The measurement of the dielectric properties shows that the electrical and dielectric properties of polypyrrole and SnO_2_/GO are successfully combined in the synthesized SGP2 nanocomposite. The measurement of the dielectric properties shows that the electrical and dielectric properties of polypyrrole and SnO_2_/GO are successfully combined in the synthesized SGP2 nanocomposite. A cyclic voltammetry analysis revealed that the SGP2 modified glassy carbon electrode-based electrochemical sensor demonstrated an excellent electrocatalytic effect for H_2_O_2_. The current amperometric sensor made from SGP2 modified GCE has a low LOD of 0.758 µM and high sensitivity of 11.69 µA mM cm^−2^ throughout a broad linear range of 0.1 mM to 0.8 mM. According to our findings, conductive polymer and metal oxide combined together could provide an efficient method of fabricating advanced and cost-effective electrochemical sensors.

## Figures and Tables

**Table 1:** Comparison of the electrochemical performance of the fabricated SGP2 modified GCE with previously published sensors for H_2_O_2_ detection.

Electrode Materials	Sensing Performance			Reference
	Sensitivity	Detection Limit	Linear Range	
MWCNT/(BIM–Cu^2+^)_n_@GCE	5 nA μM cm^−2^	0.78 µM	-	[4]
Meso-C/ZnO	0.04648 μA μM cm^−2^	6.25 μM	50 μM to 981 μM	[38]
HRP/GO-Co_3_O_4_	0.5 μA mM^−1^	2 mM	1 mM to 30 mM,	[39]
fGO–PPy–CS	-	1.95 μM	2.5–200 μM	[40]
Pt/rGO–CNT paper	1.41 μA μM cm^−2^	0.01 μM	0.1–25 μM	[41]
SGP2 modified GCE	11.69 µA mM cm^−2^	0.758 µM	0.5 mM to 7.0 mM	This Work

## Data Availability

Not applicable.

## References

[1] Kumar J.S., Murmu N.C., Kuila T. (2018). Recent trends in the graphene-based sensors for the detection of hydrogen peroxide. AIMS Mater. Sci..

[2] Halliwell B., Clement M.V., Long L.H. (2000). Hydrogen peroxide in the human body. FEBS Lett..

[3] Yusoff N., Rameshkumar P., Mehmood M.S., Pandikumar A., Lee H.W., Huang N.M. (2017). Ternary nanohybrid of reduced graphene oxide-nafion@silver nanoparticles for boosting the sensor performance in non-enzymatic amperometric detection of hydrogen peroxide. Biosens. Bioelectron..

[4] Gurusamy T., Rajaram R., Murugan R., Ramanujam K. (2022). A web of poly (bisbenzimidazolatocopper (ii)) around multiwalled carbon nanotubes for the electrochemical detection of hydrogen peroxide. New J. Chem..

[5] Xing L., Zhang W., Fu L., Lorenzo J.M., Hao Y. (2022). Fabrication and application of electrochemical sensor for analyzing hydrogen peroxide in food system and biological samples. Food Chem..

[6] Tsiafoulis C.G., Trikalitis P.N., Prodromidis M.I. (2005). Synthesis, characterization and performance of vanadium hexacyanoferrate as electrocatalyst of H_2_O_2_. Electrochem. Commun..

[7] Ma J., Song W., Chen C., Ma W., Zhao J., Tang Y. (2005). Fenton degradation of organic compounds promoted by dyes under visible irradiation. Environ. Sci. Technol..

[8] Jiang G., Tang H., Zhu L., Zhang J., Lu B. (2009). Improving electrochemical properties of liquid phase deposited TiO_2_ thin films by doping sodium dodecylsulfonate and its application as bioelectrocatalytic sensor for hydrogen peroxide. Sens. Actuators B Chem..

[9] Lou X., Zhu C., Pan H., Ma J., Zhu S., Zhang D., Jiang X. (2016). Cost-effective three-dimensional graphene/Ag aerogel composite for high-performance sensing. Electrochim. Acta.

[10] Cheng C., Huang Y., Wang N., Jiang T., Hu S., Zheng B., Yuan H., Xiao D. (2015). Facile Fabrication of Mn_2_O_3_ Nanoparticle-Assembled Hierarchical Hollow Spheres and Their Sensing for Hydrogen Peroxide. ACS Appl. Mater. Interfaces.

[11] Zhao D., Xu C. (2015). A nanoporous palladium-nickel alloy with high sensing performance towards hydrogen peroxide and glucose. J. Colloid Interface Sci..

[12] Vijeth H., Ashokkumar S.P., Yesappa L., Vandana M., Devendrappa H. (2020). Camphor sulfonic acid surfactant assisted polythiophene nanocomposite for efficient electrochemical hydrazine sensor. Mater. Res. Express.

[13] Xu F., Liu Y., Ding G., Deng M., Chen S., Wang L. (2014). Three dimensional macroporous poly(3,4-ethylenedioxythiophene) structure: Electrodeposited preparation and sensor application. Electrochim. Acta.

[14] Harlin A., Ferenets M. (2006). Introduction to conductive materials. Intelligent Textiles and Clothing.

[15] Skompska M. (2010). Hybrid conjugated polymer/semiconductor photovoltaic cells. Synth. Met..

[16] Harraz F.A. (2014). Electrochemical formation of a novel porous silicon/polypyrrole hybrid structure with enhanced electrical and optical characteristics. J. Electroanal. Chem..

[17] Najib S., Baken F., Abdullayeva N., Bahariqushchi R., Kasap S., Franzò G., Sankir M., Sankir N.D., Mirabella S., Erdem E. (2020). Tailoring morphology to control defect structures in ZnO electrodes for high-performance supercapacitor devices. Nanoscale.

[18] Vandana M., Veeresh S., Ganesh H., Nagaraju Y.S., Vijeth H., Basappa M., Davendrappa H. (2022). Graphene oxide decorated SnO_2_ quantum dots/polypyrrole ternary composites towards symmetric supercapacitor application. J. Energy Storage.

[19] Zhu H., Yang D., Yu G., Zhang H., Yao K. (2006). A simple hydrothermal route for synthesizing SnO_2_quantum dots. Nanotechnology.

[20] Lee E.J.H., Ribeiro C., Giraldi T.R., Longo E., Leite E.R., Varela J.A. (2004). Photoluminescence in quantum-confined SnO_2_ nanocrystals: Evidence of free exciton decay. Appl. Phys. Lett..

[21] Sato T. (2019). Science, Technology and Advanced Application of Supercapacitors.

[22] Hu Y., Li F., Han D., Niu L. (2014). Biocompatible Graphene for Bioanalytical Applications.

[23] Kang X., Wang J., Wu H., Aksay I.A., Liu J., Lin Y. (2009). Glucose oxidase-graphene-chitosan modified electrode for direct electrochemistry and glucose sensing. Biosens. Bioelectron..

[24] He J., Jiang Y., Peng J., Li C., Yan B., Wang X. (2016). Fast synthesis of hierarchical cuprous oxide for nonenzymatic glucose biosensors with enhanced sensitivity. J. Mater. Sci..

[25] Zhang X., Wang J., Liu J., Wu J., Chen H., Bi H. (2017). Design and preparation of a ternary composite of graphene oxide/carbon dots/polypyrrole for supercapacitor application: Importance and unique role of carbon dots. Carbon.

[26] Singh M.K., Mathpal M.C., Agarwal A. (2012). Optical properties of SnO_2_ quantum dots synthesized by laser ablation in liquid. Chem. Phys. Lett..

[27] Wazir A.H., Kundi I.W. (2016). Synthesis of Graphene Nano Sheets by the Rapid Reduction of Electrochemically Exfoliated Graphene Oxide Induced by Microwaves. J. Chem. Soc. Pak..

[28] Vishnuvardhan T.K., Kulkarni V.R., Basavaraja C., Raghavendra S.C. (2006). Synthesis, characterization and a.c. conductivity of polypyrrole/Y_2_O_3_ composites. Bull. Mater. Sci..

[29] Wang W., Hao Q., Lei W., Xia X., Wang X. (2012). Graphene/SnO_2_/polypyrrole ternary nanocomposites as supercapacitor electrode materials. RSC Adv..

[30] Kendall O., Wainer P., Barrow S., van Embden J., Gaspera E.D. (2020). Fluorine-doped tin oxidecolloidal nanocrystals. Nanomaterials.

[31] Gupta B., Kumar N., Panda K., Kanan V., Joshi S., Visoly-Fisher I. (2017). Role of oxygen functional groups in reduced graphene oxide for lubrication. Sci. Rep..

[32] Liu H., Chen Z., Wang H., Ye F., Ma J., Zheng X., Gui P., Xiong L., Wen J., Fang G. (2019). A facile room temperature solution synthesis of SnO_2_ quantum dots for perovskite solar cells. J. Mater. Chem. A Mater. Energy Sustain..

[33] Yuan Y.-G., Gurunathan S. (2017). Combination of graphene oxide-silver nanoparticle nanocomposites and cisplatin enhances apoptosis and autophagy in human cervical cancer cells. Int. J. Nanomed..

[34] Liu Y., Wang L., Yang L., Zhan Y., Zou L., Ye B. (2017). Nonenzymatic H_2_ O_2_ electrochemical sensor based on SnO_2_ -NPs coated polyethylenimine functionalized graphene. Electroanalysis.

[35] Salimi A., Hallaj R., Soltanian S., Mamkhezri H. (2007). Nanomolar detection of hydrogen peroxide on glassy carbon electrode modified with electrodeposited cobalt oxide nanoparticles. Anal. Chim. Acta.

[36] Xu C., Sun F., Gao H., Wang J. (2013). Nanoporous platinum-cobalt alloy for electrochemical sensing for ethanol, hydrogen peroxide, and glucose. Anal. Chim. Acta.

[37] Zhang T., Yuan R., Chai Y., Li W., Ling S. (2008). A Novel Nonenzymatic Hydrogen Peroxide Sensor Based on a Polypyrrole Nanowire-Copper Nanocomposite Modified Gold Electrode. Sensors.

[38] Rashed M.A., Faisal M., Harraz F.A., Jalalah M., Alsaiari M., Alsareii S.A. (2021). A highly efficient nonenzymatic hydrogen peroxide electrochemical sensor using mesoporous carbon doped ZnO nanocomposite. J. Electrochem. Soc..

[39] Asif S.A.B., Khan S.B., Asiri A.M. (2016). Electrochemical sensor for H_2_O_2_ using a glassy carbon electrode modified with a nanocomposite consisting of graphene oxide, cobalt (III) oxide, horseradish peroxidase and nafion. Microchim. Acta.

[40] Akhtar M.A., Hayat A., Iqbal N., Marty J.L., Nawaz M.H. (2017). Functionalized graphene oxide–polypyrrole–chitosan (fGO–PPy–CS) modified screen-printed electrodes for non-enzymatic hydrogen peroxide detection. J. Nanopart. Res..

[41] Sun Y., He K., Zhang Z., Zhou A., Duan H. (2015). Real-time electrochemical detection of hydrogen peroxide secretion in live cells by Pt nanoparticles decorated graphene-carbon nanotube hybrid paper electrode. Biosens. Bioelectron..

[42] Rosas-Laverde N.M., Pruna A.I., Busquets-Mataix D. (2020). Graphene Oxide-Polypyrrole Coating for Functional Ceramics. Nanomaterials.

[43] Khan S., Zulqifar, Khan T., Khan R., Khan M., Khattak S.A., Khan G. (2019). Investigation of structural, optical, electrochemical and dielectric properties of SnO_2_/GO nanocomposite. J. Mater. Sci. Mater. Electron..

[44] Sagadevan S., Podder J. (2015). Optical and electrical properties of nanocrystalline SnO_2_ thin films synthesized by chemical bath deposition method. Soft Nanosci. Lett..

[45] Peng H., Wang X., Zhao Y., Tan T., Bakenov Z., Zhang Y. (2018). Synthesis of a Flexible Freestanding Sulfur/Polyacrylonitrile/Graphene Oxide as the Cathode for Lithium/Sulfur Batteries. Polymers.

[46] Sebastian B., Thomas G.C., Manoj B. (2021). Impedance, electrical and dielectric behaviour of tin oxide nanoparticle doped with graphite, graphene oxide and reduced graphene oxide. Int. J. Electrochem. Sci..

[47] Fatima T., Sankarappa T., Ramanna R. (2016). Electrical Transport Studies in PPy/SnO_2_ Composites. Int. J. Adv. Res. Phys. Sci..

